# Supplementary material for: The structured backbone of temporal social ties
Source: Nat Commun. 2019 Jan 15;10:220. doi: 10.1038/s41467-018-08160-3 (PMC6333776; doi:10.1038/s41467-018-08160-3)
Supplement: Supplementary file 1 — Supplementary Info [file 41467_2018_8160_MOESM1_ESM.pdf]

Supplementary Information

"The structured backbone of temporal social ties"

Kobayashi et al.

Supplementary Note 1 Relationship between activity, strength and degree

Model fit

In the temporal null model, the parameters $\{a_i\}$ represent the intrinsic activities of nodes, which then determine their probability of interactions with other nodes. When considering a data set, these activities are thus not directly observable, but can be estimated by a maximum likelihood method as described in the Methods section. Given its definition, the estimated activity of a node in a temporal network is expected to be related to the total number of snapshot edges involving that node, namely its strength. Supplementary Figure 1a shows indeed that the estimated activity is proportional to the empirical strength. In Supplementary Figure 1b we also show that the empirical strength is correctly predicted by the value obtained in the model, namely $\tau\sum_{j\neq i} u(a_i^*, a_j^*)$. Supplementary Figure 1c finally compares the total numbers of snapshot edges in the data ($M = \sum_{i<j} m_{ij}$) and the model $M^* = \tau\sum_{i<j} u(a_i^*, a_j^*)$. The almost perfect fit between the estimated and empirical values of the strength and of the total numbers of edges indicates that the maximum likelihood estimation of the activity vector works well for a wide range of temporal-network data.

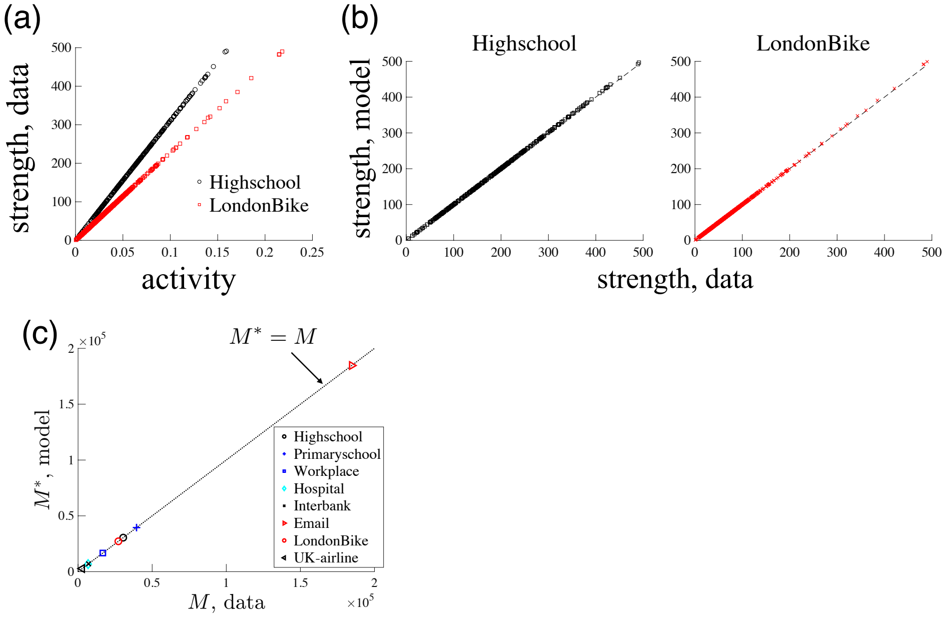

Supplementary Figure 1. Comparison between the model and the data. (a) Empirical strength vs. estimated activity. Each dot represents a node. Activity is estimated using the temporal null model described in the main text. The strength denotes the total number of snapshot edges emanating from a node. (b) Theoretical vs. empirical strength. The dashed line denotes the 45-degree line. The theoretical strength of node i with estimated activity a_i^* is given by $\tau\sum_{j\neq i} u(a_i^*, a_j^*)$. (c) Comparison between the total number of snapshot edges in the data and the model.

Node degree and significant ties

Our temporal null model does not consider the node aggregate degree as an argument of the interaction probability u (the aggregate degree is the number of neighbors in the aggregate network, i.e., number of distinct nodes with whom a node has had at least one interaction). A first motivation for this is to avoid dealing with a more complex model and a large number of additional parameters.

For completeness, we examine here the correlations between activity, degree and significant ties. First, Supplementary Figure 2 shows that the degree has a strong positive correlation with the estimated activity in all data sets. This suggests that the estimated activities carry some information on the aggregate degree.

We also show in Supplementary Figure 3 that there actually exists a weak negative correlation between activity and the fraction of significant edges emanating from a node, represented by K^{sig}/K, where K^{sig} denotes the number of significant edges. This would tend to show the relevance of enriching the null model with degree information. However, we do not find any significant correlation between K and K^{sig}/K *for a given level of activity*. This indicates that once activity is taken into account, and as it is already strongly correlated with the degree, node degree would not be more informative in predicting the likelihood of having significant ties.

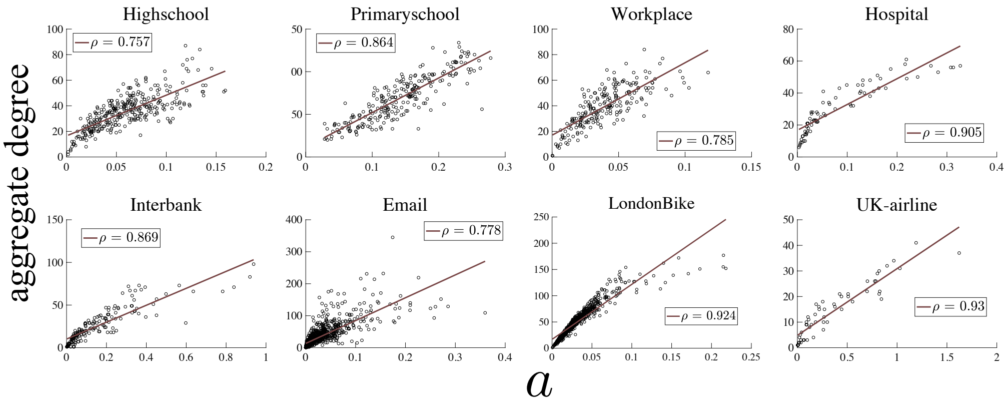

Supplementary Figure 2. Aggregate degree vs. activity. Activities are estimated using the temporal null model. ρ denotes the Pearson correlation coefficient. The aggregate degree (i.e., the number of distinct neighbors) has a positive correlation with the estimated activity.

Time-varying matching probability

In constructing the temporal fitness model, it is possible to take into account the possibility that the probability of an interaction between two nodes can vary over time even when individuals' intrinsic activities $\{a_i\}$ are constant. This can happen when, for example, a school schedule has a certain rhythm (e.g., lunch time, class schedule, etc), or due to circadian or weekly rhythms. The probability u for the existence of an interaction between two nodes i and j at time t is then given as

$$u(a_i, a_j, t) \equiv a_i a_j \xi(t), \;\; t = 1, \ldots, \tau, \tag{1}$$

where $\xi(t)$ denotes a time-varying parameter. We assume that there is no correlation between the values of ξ at different times, and the interaction probabilities are independent across time intervals.

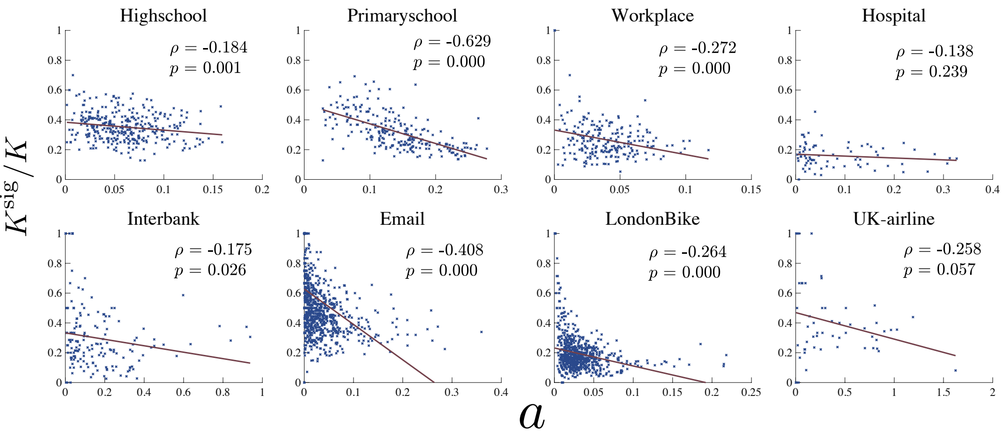

Supplementary Figure 3. Negative correlation between the share of significant ties and activity. $K^{\rm sig}$ denotes the number of significant ties of a node (here at $\alpha = 0.01$) while K is its aggregate degree. Each dot denotes a node, and the solid line shows a linear regression. ρ and p denote the Pearson correlation coefficient and its p-value, respectively. Nodes with lower activities are slightly more likely to have significant ties.

The joint probability function for a certain temporal network $\{A_t\}$ is obtained as

$$p(\{A_t\}|\boldsymbol{a},\boldsymbol{\xi}) = \prod_{t=1}^{\tau}\prod_{i,j:i\neq j} u(a_i,a_j,t)^{A_{ij,t}}(1-u(a_i,a_j,t))^{1-A_{ij,t}}, \tag{2}$$

where $A_{ij,t}$ is the (i,j) element of the adjacency matrix in time interval t, denoted by A_t, and $\boldsymbol{\xi} \equiv (\xi(1),\ldots,\xi(\tau))^\top$. The log-likelihood function is thus given by

$$\begin{aligned}\mathscr{L}(\boldsymbol{a},\boldsymbol{\xi}) &= \log p(\{A_t\}|\boldsymbol{a},\boldsymbol{\xi})\\ &= \sum_{t=1}^{\tau}\sum_{i,j:i\neq j}\left[A_{ij,t}\log\left(a_i a_j \xi(t)\right) + (1-A_{ij,t})\log\left(1-a_i a_j\xi(t)\right)\right],\end{aligned} \tag{3}$$

The maximum-likelihood estimate of $(\boldsymbol{a},\boldsymbol{\xi})$ is the solution for the following $N+\tau-1$ equations:

$$H_i^{\rm act}(\boldsymbol{a}^*,\boldsymbol{\xi}^*) \equiv \sum_{t=1}^{\tau}\sum_{j:j\neq i}\frac{A_{ij,t}-a_i^*a_j^*\xi^*(t)}{1-a_i^*a_j^*\xi^*(t)} = 0,\ \ i = 1,\ldots,N, \tag{4}$$

$$H_t^{\rm time}(\boldsymbol{a}^*,\boldsymbol{\xi}^*) \equiv \sum_{i,j:j\neq i}\frac{A_{ij,t}-a_i^*a_j^*\xi^*(t)}{1-a_i^*a_j^*\xi^*(t)} = 0,\ \ t = 2,\ldots,\tau, \tag{5}$$

These first-order conditions are obtained by differentiating the log-likelihood function Supplementary Equation (3) with respect to a_i for $i = 1,\ldots N$ and $\xi(t)$ for $t = 2,\ldots,\tau$. For $t = 1$, $\xi(1)$ is normalized as one since otherwise there would arise a linear dependency between the optimality conditions and therefore the solution would be indeterminate. This reflects the fact that any combination of $\hat{a}_i$, $\hat{a}_j$ and $\hat{\xi}(t)$ would satisfy the optimality conditions if $a_i^*a_j^* = c\cdot\hat{a}_i\hat{a}_j$ and $\xi^*(t) = \hat{\xi}(t)/c$. In solving the nonlinear Supplementary Equations (4) and (5), the initial values for a_i and $\xi(t)$ are set as $a_i = \sum_{j:j\neq i}(m_{ij}/\tau)/\sqrt{2\sum_{i<j}m_{ij}/\tau}$ and 0.999, respectively.

Under the null model with a time-varying term, the average number of contacts between i and j over τ periods is given by

$$\lambda_{ij} \equiv \sum_{t=1}^{\tau} u(a_i, a_j, t), \; \forall\, i, j. \tag{6}$$

Thus, the number of contacts obeys a Poisson binomial distribution with mean λ_{ij} and variance $\sigma_{ij} \equiv \sum_{t=1}^{\tau}(1 - u(a_i, a_j, t))u(a_i, a_j, t)$. Since an exact functional form for a Poisson binomial distribution is intractable, we approximate the distribution of $\{m_{ij}\}$ with a Poisson distribution[1–3]:

$$f(m_{ij}|\boldsymbol{a}, \boldsymbol{\xi}) \approx \frac{\lambda_{ij}^{m_{ij}} e^{-\lambda_{ij}}}{m_{ij}!} \equiv \widetilde{f}(m_{ij}|\boldsymbol{a}, \boldsymbol{\xi}), \tag{7}$$

where the error bound is given by Le Cam's theorem[1–3]:

$$\sum_{m_{ij}=0}^{\infty} \left| f(m_{ij}|\boldsymbol{a}, \boldsymbol{\xi}) - \frac{\lambda_i^{m_{ij}} e^{-\lambda_{ij}}}{m_{ij}!} \right| < \frac{2(1 - e^{-\lambda_{ij}})}{\lambda_{ij}} \sum_{t=0}^{\tau} u(a_i, a_j, t)^2, \; \forall\, i, j. \tag{8}$$

We use Supplementary Equation (7) in testing the significance of edge (i, j) for a given observation m_{ij}^{o}.

A comparison between the models with and without time-varying parameter is shown in Supplementary Figure 4. It shows that the test results are almost identical between the two null models for most data sets; the numbers and the degree of overlap of identified significant ties suggests that the introduction of a time-varying parameter for capturing an activity rhythm does not affect the results shown in the main text.

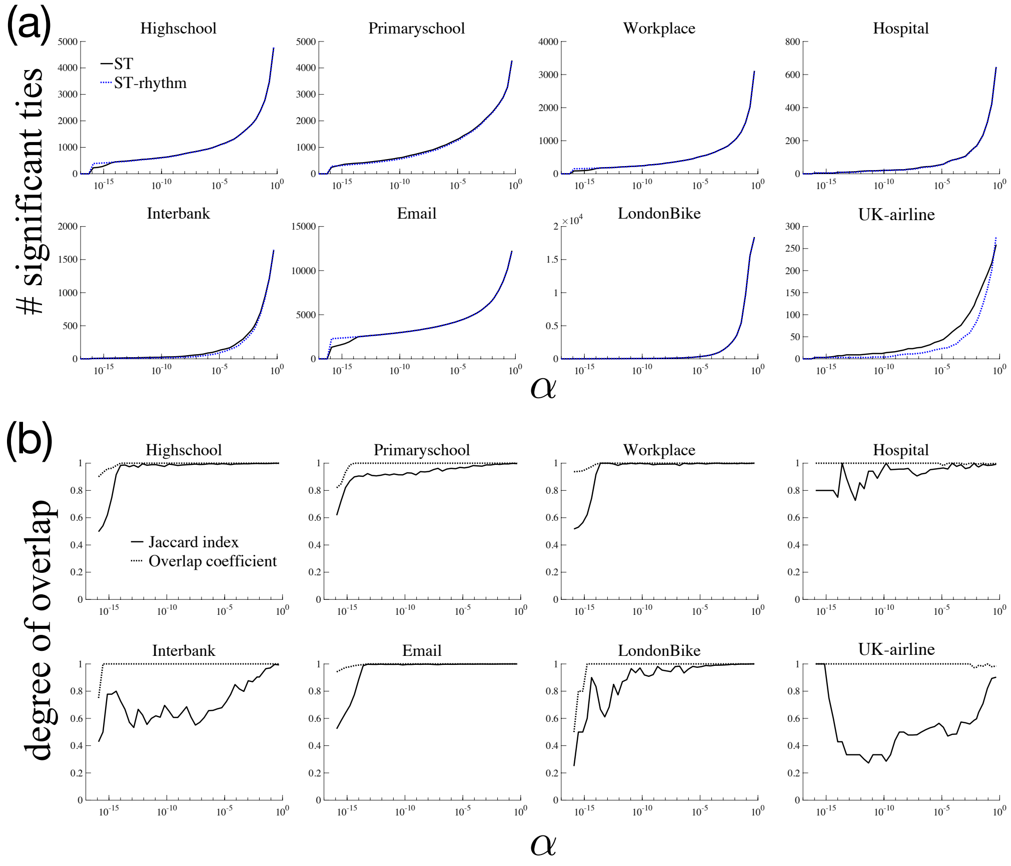

Supplementary Figure 4. Comparison between constant and time-varying interaction probabilities. (a) Number of significant ties vs. significance level α. ST-rhythm denotes the ST filter with a time-varying parameter. (b) Jaccard index and overlap coefficient for quantifying the overlap between the lists of significant ties detected by the two null models, vs. α. The overlap coefficient, or Szymkiewicz–Simpson coefficient, is defined by $S(I_{\text{ST}}, I_{\text{ST-rhythm}}) \equiv |I_{\text{ST}} \cap I_{\text{ST-rhythm}}| / \min(|I_{\text{ST}}|, |I_{\text{ST-rhythm}}|)$. The fact that it remains equal or very close to 1 indicates that, even in the few cases where the Jaccard index is not very large, the set of significant ties identified by the ST-rhythm is included in the set identified by the ST filter.

Supplementary Note 2 Backboning methods for static networks

We recall here two well-known ways to assign a significance to edges and build backbones for static weighted networks. In our context, it can be done by first aggregating the temporal network on the available time-window, obtaining a network where the degree of a node is given by its number of distinct neighbors, the weight of an edge is total interaction time between two nodes, and the strength of an edge is given by $s_i = \sum_{j,t} A_{ij,t}$.

Disparity filter

The Disparity (DP) filter[4] is a filtering algorithm to classify the edges of a static weighted network into significant and insignificant ones. The DP filter uses only local information: the weight of an edge, ω_{ij}, the nodal degree, k_i, and the strength, s_i. The idea is that if node i has no specific relationship with its neighbors, then its strength (i.e., sum of weights) is distributed uniformly at random on the k_i edges incident to it. The authors of[4] show that the link between i and j is regarded as significant at filtering level α, if it satisfies the following condition:

$$1-(k_i-1)\int_0^{p_{ij}}(1-x)^{k_i-2}dx < \alpha, \tag{9}$$

where $p_{ij} = \omega_{ij}/s_i$. The LHS of Supplementary Equation (9) represents the p-value for the null hypothesis that the edge weights are distributed uniformly at random. In fact, the significance of edge between i and j is not necessarily identical to that between j and i even for an undirected network[4]. Therefore, one needs to test the significance of "two edges" (i, j) and (j, i) independently, and then the (undirected) edge is regarded as significant if at least one of the two "edges" satisfies the criterion (9).

ECM filter

The ECM (enhanced configuration model) filter[5] is developed based on the idea that statistically significant edges are the ones whose presence cannot be explained by random chance. More specifically, the entropy-maximizing random matching probabilities are calculated with the ECM in which edge weights are distributed at random as uniformly as possible subject to two constraints: $\langle\vec{k}\rangle = \vec{k}^*$ and $\langle\vec{s}\rangle = \vec{s}^*$, where x^* denotes the empirical value of variable x. Gemmetto et al.[5] show that the p-value for edge (i, j) is then given by

$$\gamma^*_{ij} = p^*_{ij}(y^*_i y^*_j)^{w^*_{ij}-1}, \tag{10}$$

where

$$p^*_{ij} = \frac{x^*_i x^*_j y^*_i y^*_j}{1-y^*_i y^*_j + x^*_i x^*_j y^*_i y^*_j}, \tag{11}$$

and x^*_i and y^*_i represent *hidden variables* (or auxiliary variables)[6,7] that solve the following conditions:

$$k^*_i = \sum_{j\neq i} \frac{x_i x_j y_i y_j}{1-y_i y_j + x_i x_j y_i y_j} \quad \forall i, \tag{12}$$

$$s^*_i = \sum_{j\neq i} \frac{x_i x_j y_i y_j}{(1-y_i y_j)(1-y_i y_j + x_i x_j y_i y_j)} \quad \forall i. \tag{13}$$

One needs to solve a system of $2N$ nonlinear equations to obtain $\vec{x}^*$ and $\vec{y}^*$. In fact, $-\ln x_i$ $(-\ln y_i)$ corresponds to a Lagrange multiplier associated with the constraint $\langle k_i\rangle = k^*_i$ $(\langle s_i\rangle = s^*_i)$. The backbone of a weighted network with significance level α is the network consisting only of edges $(i, j) \in \{(i, j) : \gamma^*_{ij} < \alpha\}$. Our implementation for the calculation of Supplementary Equations (12) and (13) is based on the "Max & Sam" method proposed in[8], and the MATLAB code is available from[9].

Supplementary Note 3 Generating synthetic temporal networks

Synthetic networks with a known set of significant ties

To examine the detectability of strong ties in a controlled setting, we generate synthetic temporal networks in which the fraction of such ties is set a priori. The network-generating procedure is given by:

1. We consider N nodes, each with an intrinsic activity drawn from a Beta distribution, $a_i' \sim \mathrm{Beta}(1, 10)$.
2. We generate a temporal network in the time-window $[0, T'-1]$: at each time-step, each pair of nodes (i, j) is connected with probability $a_i' a_j', \forall i \neq j$. This yields a sequence of T' undirected and unweighted networks, $\widehat{A}(0), \widehat{A}(1), \ldots, \widehat{A}(T'-1)$.
3. We will consider as "data set" the last T time-steps, i.e., the time-window $[T'-T, T'-1]$. Among the pairs with at least one interaction in this time-window, we randomly select 20% as having "strong" ties.
4. We construct T' new networks $A(0), \cdots A(T'-1)$ from $\widehat{A}(0), \cdots \widehat{A}(T'-1)$ by adding interactions among the strong ties as follows (for the other ties, we set $A_{ij}(t) = \widehat{A}_{ij}(t)$ for $t = 1, \cdots, T'-1$): for each strong tie (i, j), we initialize $A_{ij}(0) = \widehat{A}_{ij}(0)$ and we repeat for $t = 1, \cdots, T'-1$

   - if $\widehat{A}_{ij}(t) = 1$, we keep $A_{ij}(t) = 1$;
   - if $\widehat{A}_{ij}(t) = 0$ and $A_{ij}(t-1) = 1$, i.e., if i and j are ending an interaction, we set $A_{ij}(t) = 0$ with probability $h_{ij}(t)$ and $A_{ij}(t) = 1$ with probability $1 - h_{ij}(t)$, where $h_{ij}(t) = \frac{1}{1+b \cdot D_{ij}(t-1)}$ and $D_{ij}(t-1)$ denotes the number of consecutive periods up to $t-1$ in which i and j are in interaction. In other terms, the longer i and j have been interacting, the more probable it is that they continue to interact[10].

   The non-negative parameter b tunes the strength of the strong ties; $b = 0$ corresponds to a situation in which there are no strong ties.
5. We use the last T time-steps as our synthetic data set: we create a sequence of $\tau = \lfloor T/\Delta \rfloor$ snapshots by aggregating over time-windows of Δ consecutive time-steps. In each snapshot, the aggregate edges are binarized for the ST filter and weighted by the number of interactions for the ECM-R filter. For the DP and ECM filters, a weighted network is created by aggregating over the T snapshots.

We set $N = 300$, $\Delta = 10$, $T = 300$ and $b = 5$. A sequence of $T' = 3000$ networks is generated in each run and the initial 2700 periods are discarded (i.e., $T = T' - 2700 = 300$). The results shown in the main text are obtained with 100 realizations of such synthetic networks.

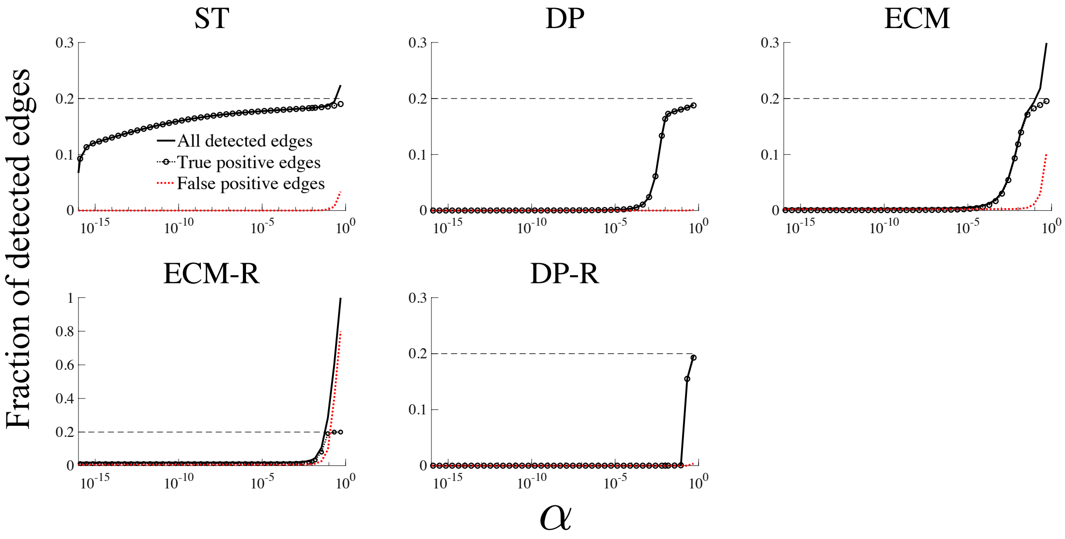

Supplementary Figure 5. Fraction of detected edges vs. significance level for each filtering algorithm implemented on synthetic networks. The ground-truth fraction of strong edges is 0.2 (denoted by a black dashed line). The black solid lines denote the mean fraction of detected edges using the method given at the top of each panel. The dotted lines with circles illustrate the mean fraction of detected edges excluding false positive ones; i.e., the fraction contains only those edges that are genuinely strong in the synthetic data. Dotted red lines denote the mean fraction of detected significant ties that are not strong ties in the synthetic data, i.e., false positives. See section Supplementary Note 3 for details about the generation of synthetic networks.

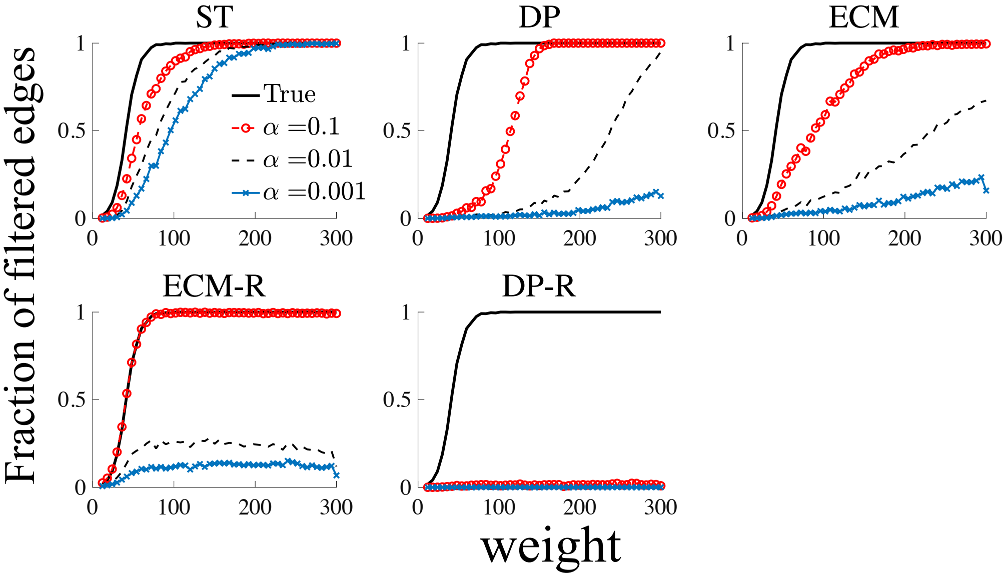

Supplementary Figure 6. Average fraction of filtered edges against weights. For each weight bin, the share of filtered edges is calculated, averaged over 100 synthetic networks generated as in section Supplementary Note 3. Solid line denotes the ground-truth fraction of significant ties among the edges whose weights lie within a given bin.

Synthetic networks with both significant and weight reinforced ties

We here generate synthetic temporal networks with two types of artificially reinforced ties, in order to highlight how different filters detect different types of ties. The first type corresponds to significant ties as in the previous section Supplementary Note 3. The second type is built to be detected by the static filters and their simple generalizations but not by our ST filter. More precisely, the network-generating procedure is given by (the first steps are as in the previous section):

1. We consider N nodes, each with an intrinsic activity drawn from a Beta distribution, $a'_i \sim \mathrm{Beta}(1,10)$.

2. We generate a temporal network in the time-window $[0, T'-1]$: at each time-step, each pair of nodes (i, j) is connected with probability $a'_i a'_j, \forall i \neq j$. This yields a sequence of T' undirected and unweighted networks, $\widehat{A}(0), \widehat{A}(1), \ldots, \widehat{A}(T'-1)$.

3. We will consider as "data set" the last τ time-steps, i.e., the time-window $[T'-\tau, T'-1]$. Among the pairs with at least one interaction in this time-window, we randomly select 10% as having (dynamic) significant ties (as in section Supplementary Note 3) and another 10% as having "weight reinforced" ties.

4. We construct networks with significant ties as explained in step 4 of section Supplementary Note 3. On top of that, for each snapshot, we consider each weight reinforced tie: if the tie is present in the snapshot, it is assigned the weight $w_{\mathrm{reinforced}}$ (nothing is done if the tie is not present, so the sequence of snapshots in which the tie is present is not altered). The other present ties are assigned the weight 1 in the snapshot. We set $w_{\mathrm{reinforced}} = \tau$.

5. For the ST filter, the edges in each snapshot are binarized. For the DP and ECM filters, an aggregate network is created by summing up all the edge weights over the τ snapshots.

A schematic view of the procedure is presented in Fig. 7. For our numerical experiments, we set $N = 300$, $\tau = 30$ and $b = 5$. A sequence of $T' = 3000$ networks is generated in each run and the initial 2970 periods are discarded (i.e., $\tau = T' - 2970 = 30$). The average fraction of detected ties of each type shown in Fig. 8 is computed over 100 simulations. The figure shows that the ST filter detects efficiently and on a very broad range of significance values the dynamic significant ties, but does not detect any of the weight reinforced ties. On the other hand, the static filters detect both types in the same fashion, and the ECM-R and DP-R detect the weight reinforced edges on a broader range of significance values than the significant ties.

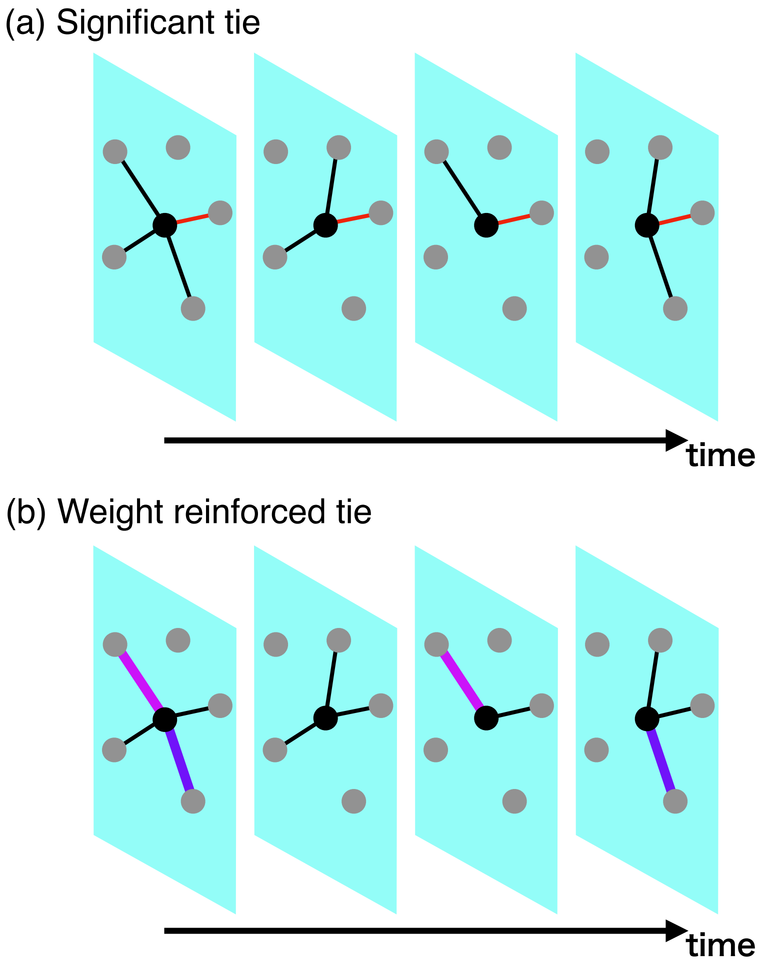

Supplementary Figure 7. Schematic description of the procedure. (a) Sketch of significant ties. For a pair to be regarded as having a significant tie, there must be sufficiently many snapshot edges between the nodes (so that it cannot be explained by random chance), but the edges do not need to have large weights (as in the case of the tie with red lines). (b) For the pairs with reinforced weights, each snapshot edge is assigned a large weight (blue and light purple lines).

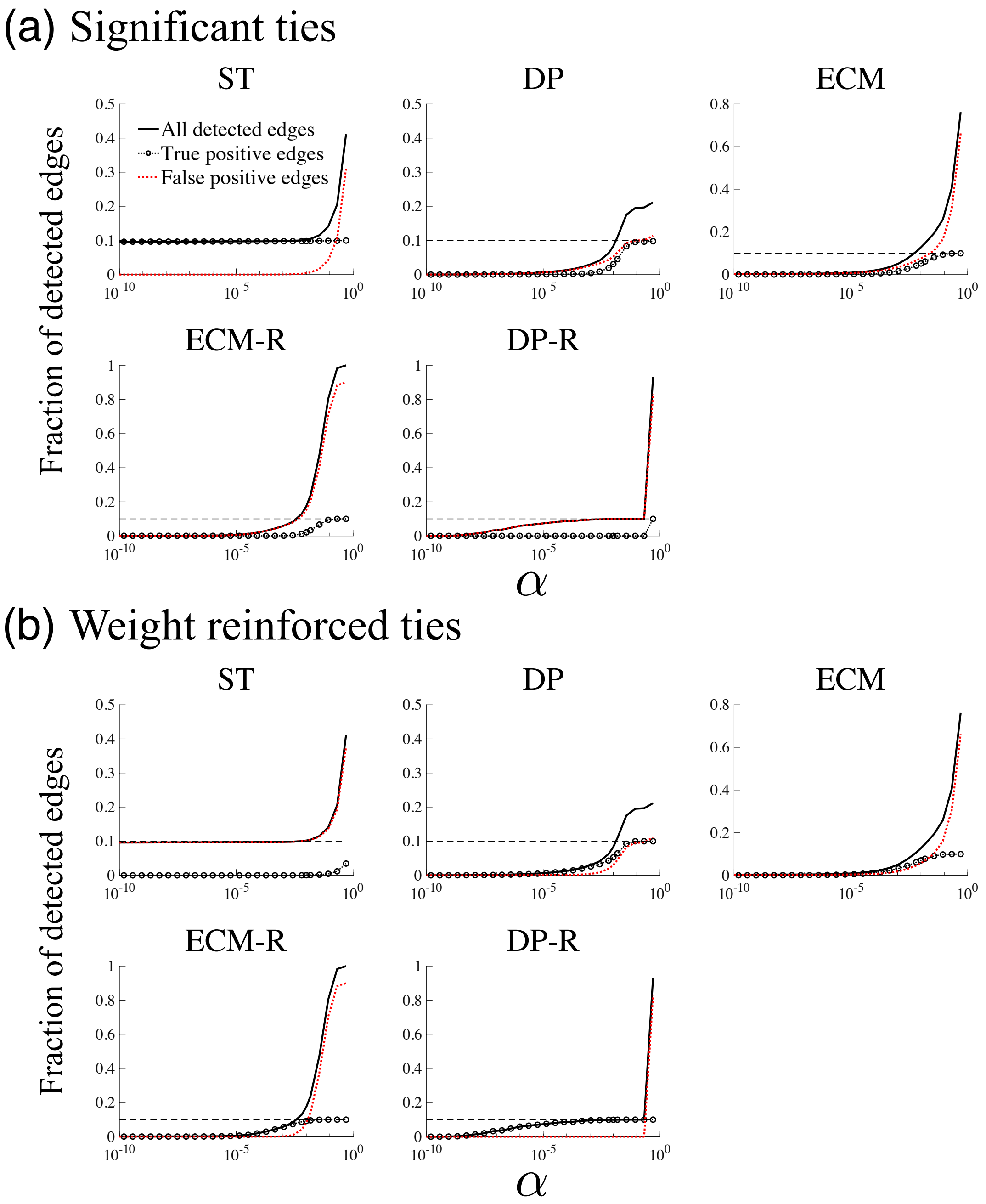

Supplementary Figure 8. Fraction of detected edges vs. significance level on synthetic networks with both (dynamic) significant ties and weight reinforced ties. The ground-truth fractions are both 0.1 (black dashed line). (a) Detectability of (dynamic) significant ties. (b) Detectability of weight reinforced ties. In each case, black solid lines denote the mean fraction of all detected edges while dashed lines with circles represent the mean fraction of correctly detected significant ties (resp. weight reinforced ties) excluding false positive ones; i.e., the fraction contains only those edges that are genuinely significant ties (resp. weight reinforced ties). Red dotted illustrate the mean fraction of detected edges that are not significant ties (resp. weight reinforced ties).

Supplementary Note 4 Definitions of ROC curve and AUC

The receiver operating characteristic (ROC) curve is a plot of true positive rates against false positive rates for different cutpoints of a test statistic. In our context, we want to know how well the significance of an edge can predict whether that edge is an intra-community edge. For this purpose, we use the p-value of an edge in a given filtering test as a measure of edge significance. That is, different points on an ROC curve denote different cutoff levels of p-values for a given filtering method (Supplementary Figure 9). For the ST filter presented in the main text, the p-value of an edge $i-j$ is simply $1-G(m_{ij}^c|a_i^*,a_j^*)$.

For a given p_0, we consider therefore as True Positives (TP) the edges that have a p-value lower than p_0 (i.e., are considered significant) and are intra-community edges. False Positives (FP) are instead the inter-community edges with a p-value lower than p_0. Similarly, True Negatives (TM) are edges with p-value larger than p_0 and inter-community, and False Negatives (FN) the intra-community edges with p-value larger than p_0. The false positive rate is given by $FP/(TN+FP)$ and the true positive rate by $TP/(TP+FN)$.

The area under the ROC curve (AUC) quantifies the goodness of the p-values for the task of predicting intra-community edges. If the p-value of a filtering method perfectly distinguishes between intra- and inter-community edges (i.e., higher p-values indicate inter-community edges), then the value of AUC will be one. If the p-value is instead a poor indicator so that it is not different from a random prediction, then the AUC will be close to 0.5 (i.e., the ROC curve is then be a straight line joining the points $(0,0)$ and $(1,1)$). We show the ROC curves and a comparison of the AUCs for different data sets in Supplementary Figure 9 and Fig. 5c in the main text.

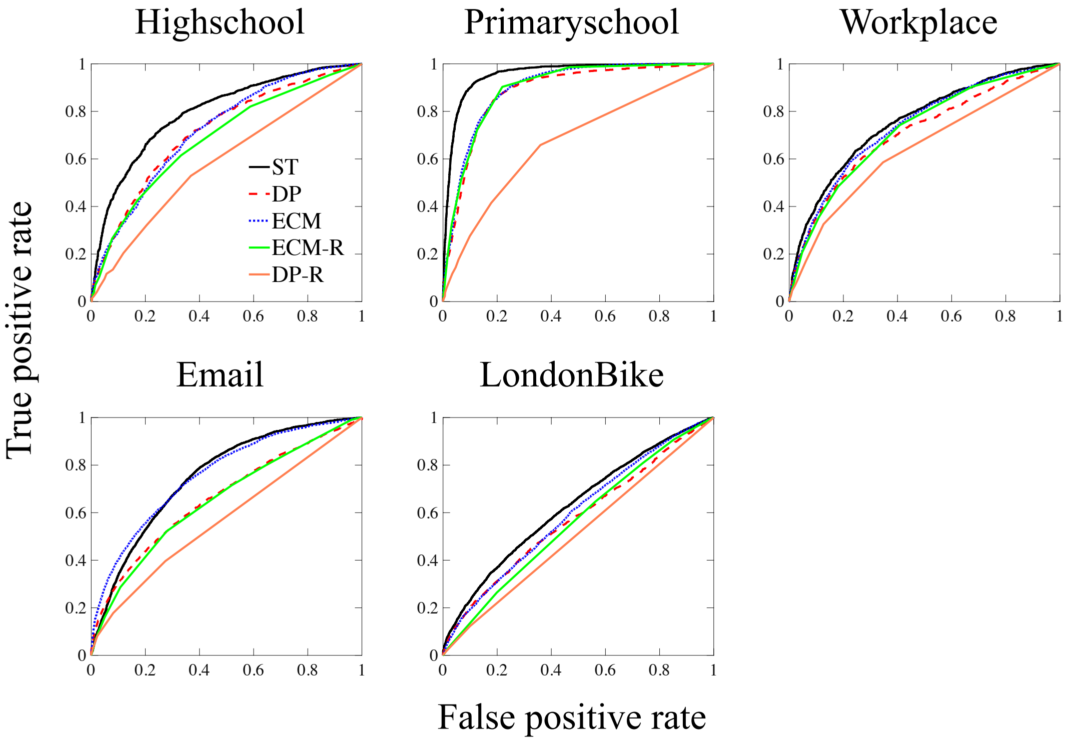

Supplementary Figure 9. ROC curve for the detection of intra-community edges for the data sets with a community structure, for the filters considered in the main text: Disparity filter (DP), DP-R, Enhanced Configuration model (ECM), ECM-R and ST filter.

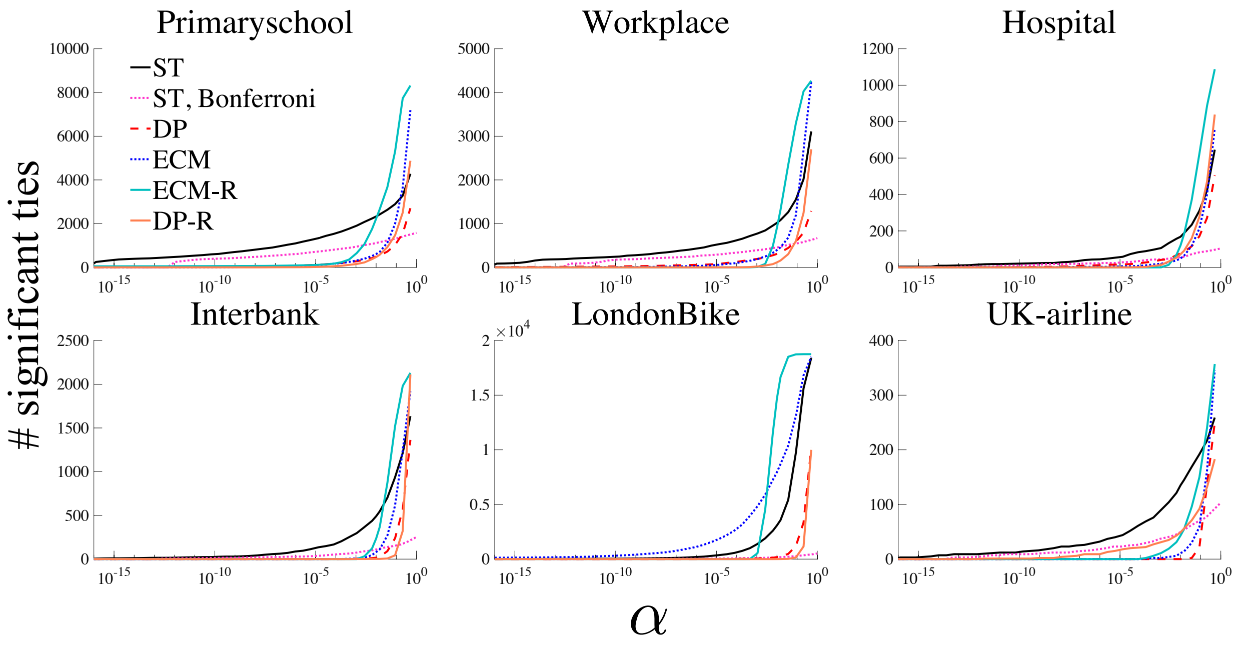

Supplementary Figure 10. Number of significant ties vs. α, for the different filters considered.

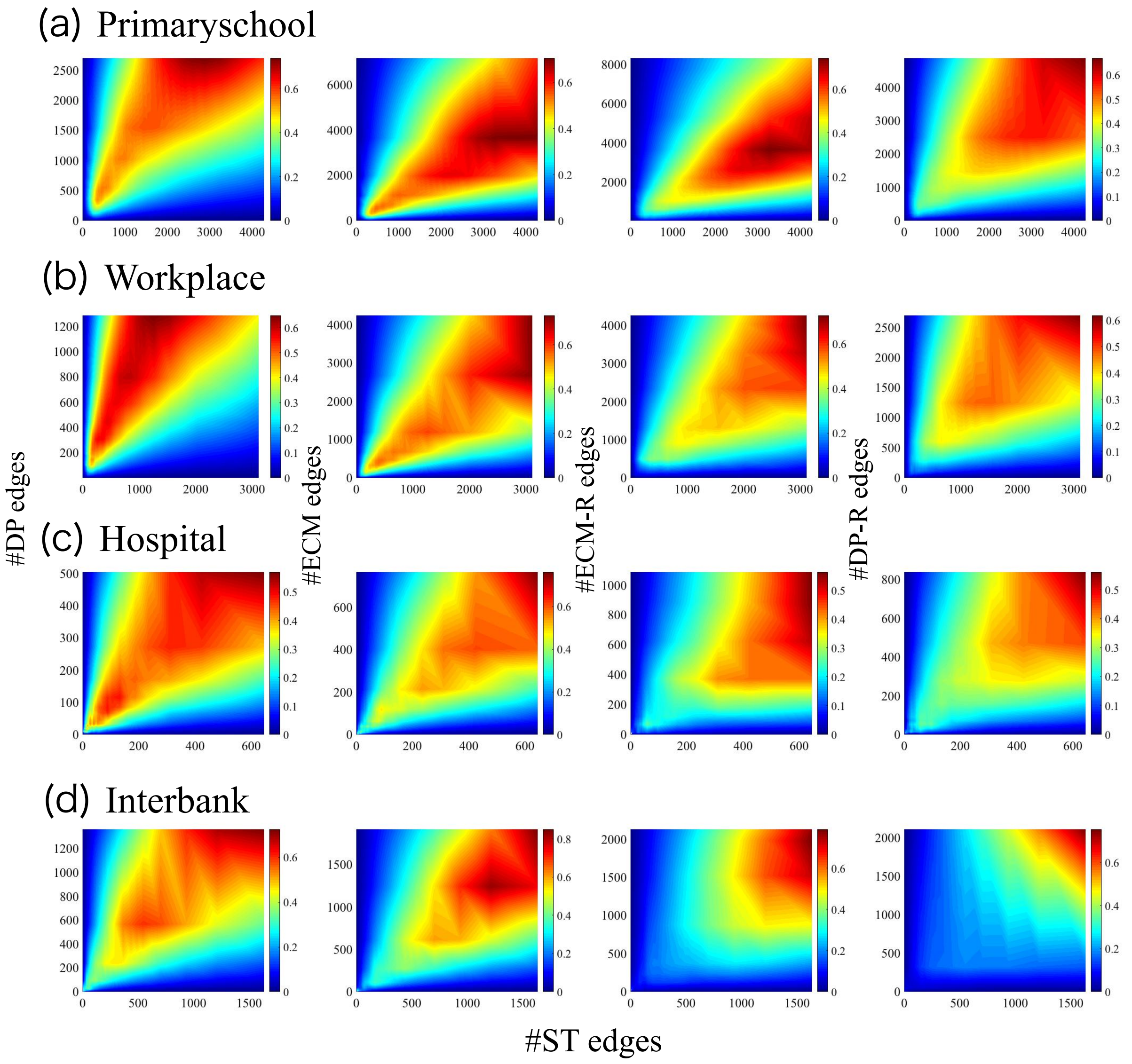

Supplementary Figure 11. Jaccard index of the similarity between the backbones obtained by various filtering methods, vs. the number of edges retained in each case. Jaccard index is defined by $J(I^{\alpha}_{\mathrm{ST}}, I^{\alpha'}_{\mathrm{x}}) = |I^{\alpha}_{\mathrm{ST}} \cap I^{\alpha'}_{\mathrm{x}}| / |I^{\alpha}_{\mathrm{ST}} \cup I^{\alpha'}_{\mathrm{x}}|$, where x denotes the filtering method (DP, ECM, ECM-R, DP-R).

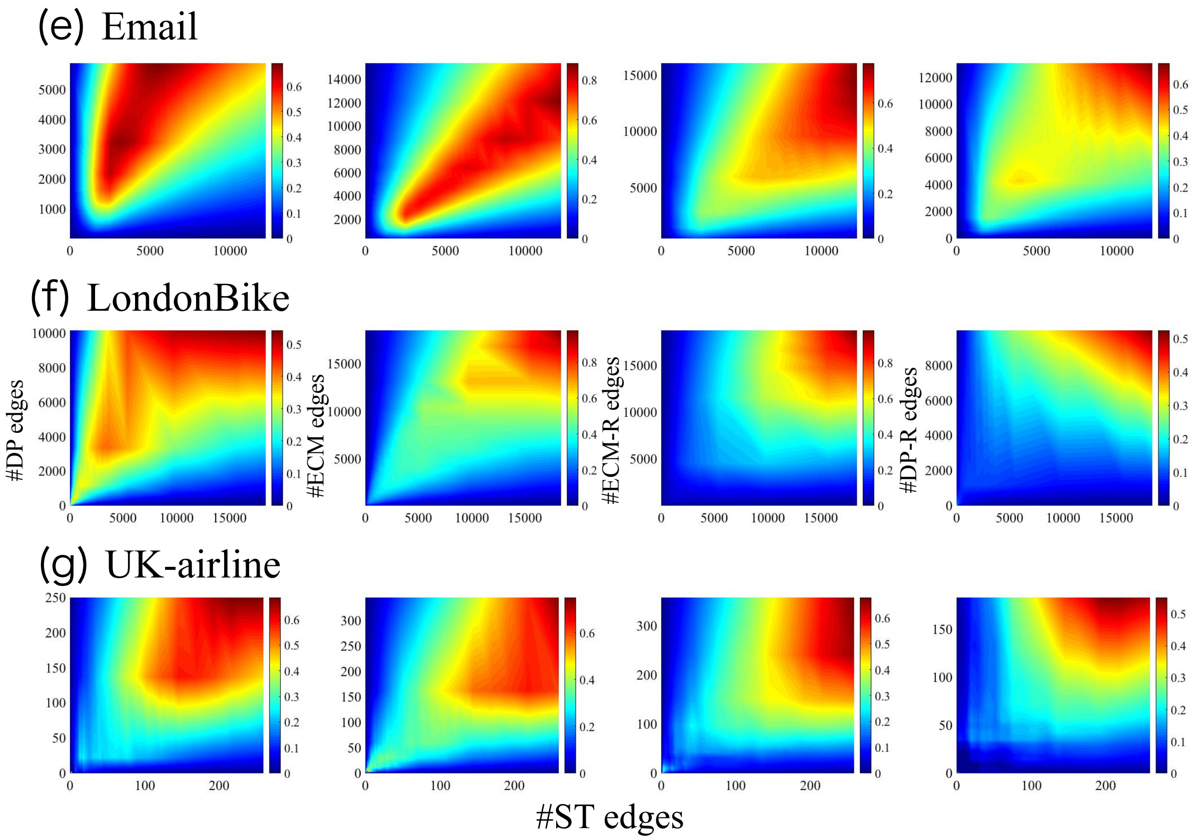

(e) Email
(f) LondonBike
(g) UK-airline
#DP edges
#ECM edges
#ECM-R edges
#DP-R edges
#ST edges

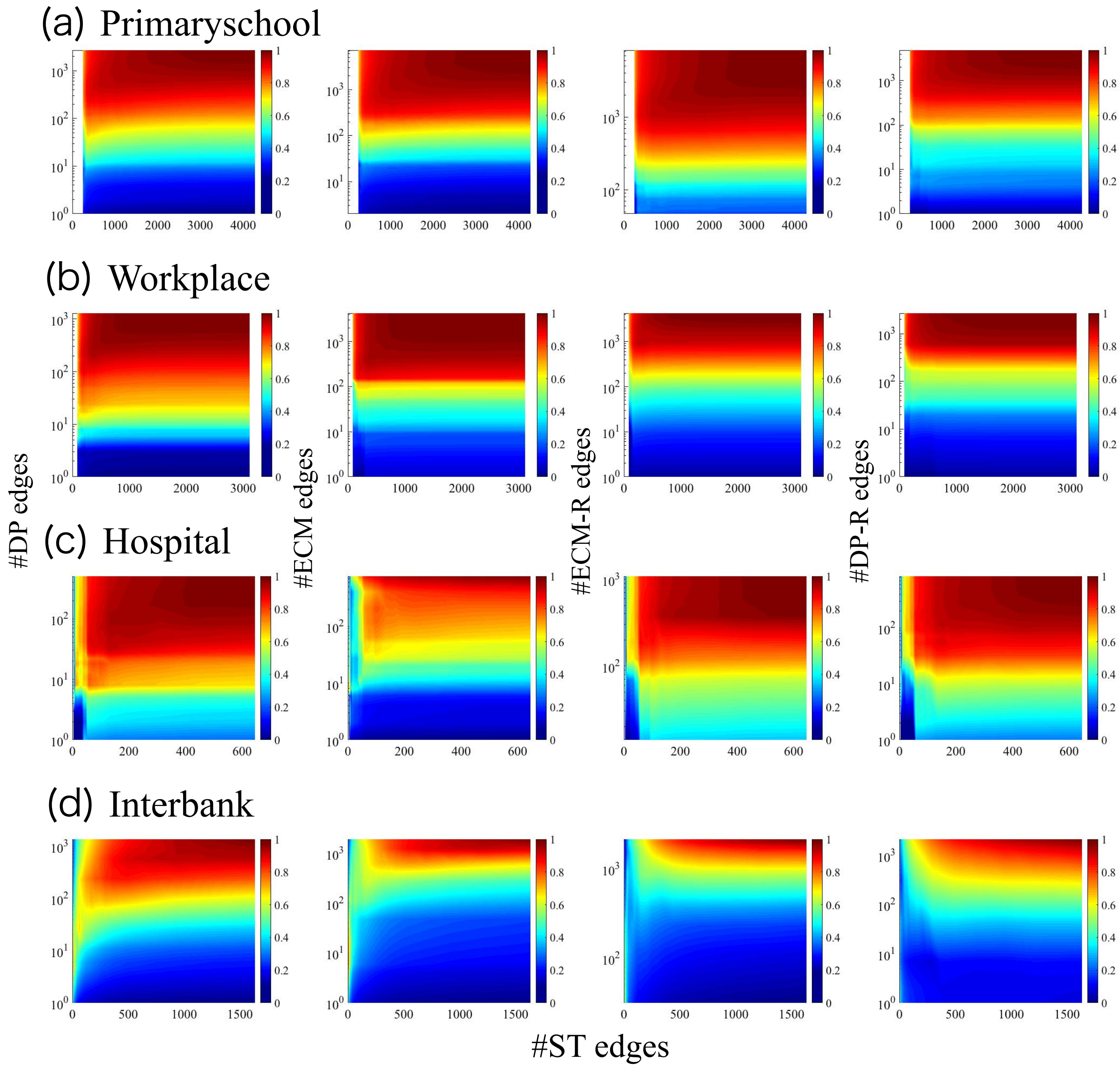

Supplementary Figure 12. Weighted measure of the similarity between the backbones obtained by various filtering methods, vs. the number of edges retained in each case. Here we use the cosine similarity between the weights of the edges retained by two methods, defined as $\sigma(\mathrm{x},\mathrm{x}') = \frac{\sum_{i<j} w_{ij}^{\mathrm{x}} w_{ij}^{\mathrm{x}'}}{\sqrt{\sum_{i<j}\left(w_{ij}^{\mathrm{x}}\right)^2}\sqrt{\sum_{i<j}\left(w_{ij}^{\mathrm{x}'}\right)^2}}$, where x encodes both the filtering method (ST, DP, ECM, ECM-R, DP-R) and the significance level α and the sums run on the pairs of nodes present in the backbones.

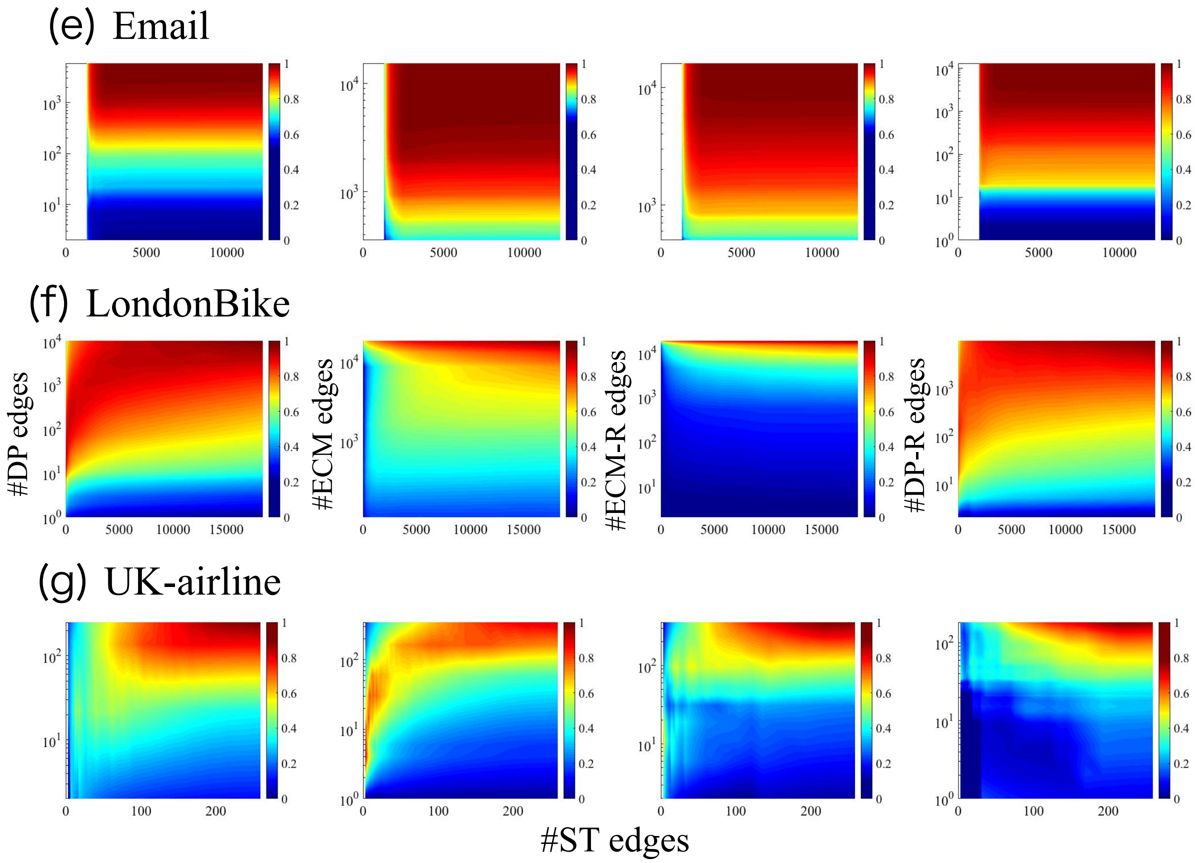
(e) Email
(f) LondonBike
(g) UK-airline
#DP edges
#ECM edges
#ECM-R edges
#DP-R edges
#ST edges
0
5000
10000
15000
100
200
0.2
0.4
0.6
0.8
1

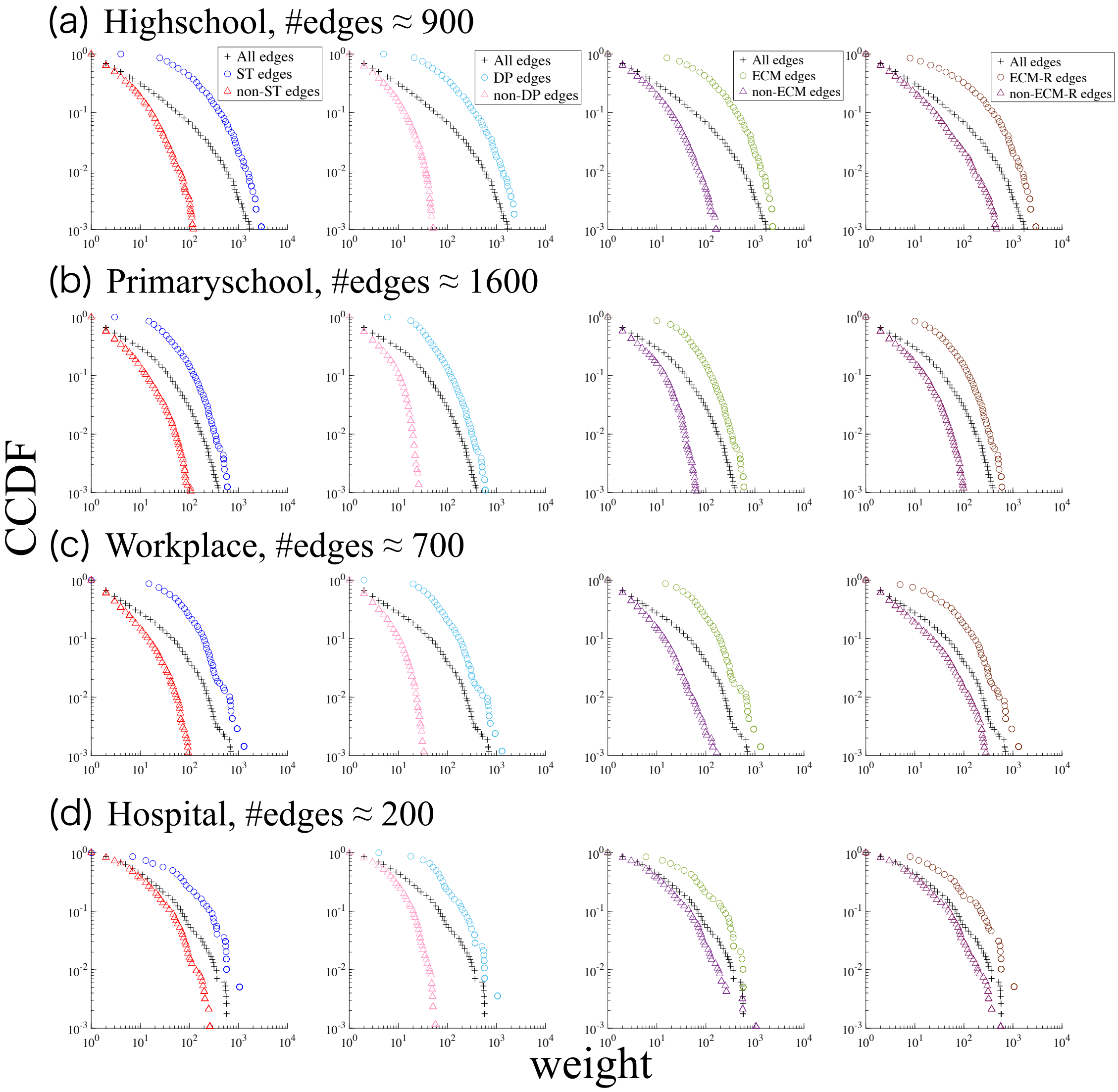

Supplementary Figure 13. Weight distributions of significant and non-significant edges for a fixed number of edges, for the ST filter (first column), the DP filter (second column), the ECM filter (third column) and the ECM-R filter (fourth column). In panels (a)–(g), the weights represent the total number of interactions. In panel (h), the total number of passengers is used as weight, since the number of interactions is quite small (namely, the number of flights is at most 14). DP is in general closer to a simple thresholding than the other filters (see discussion in the main text).

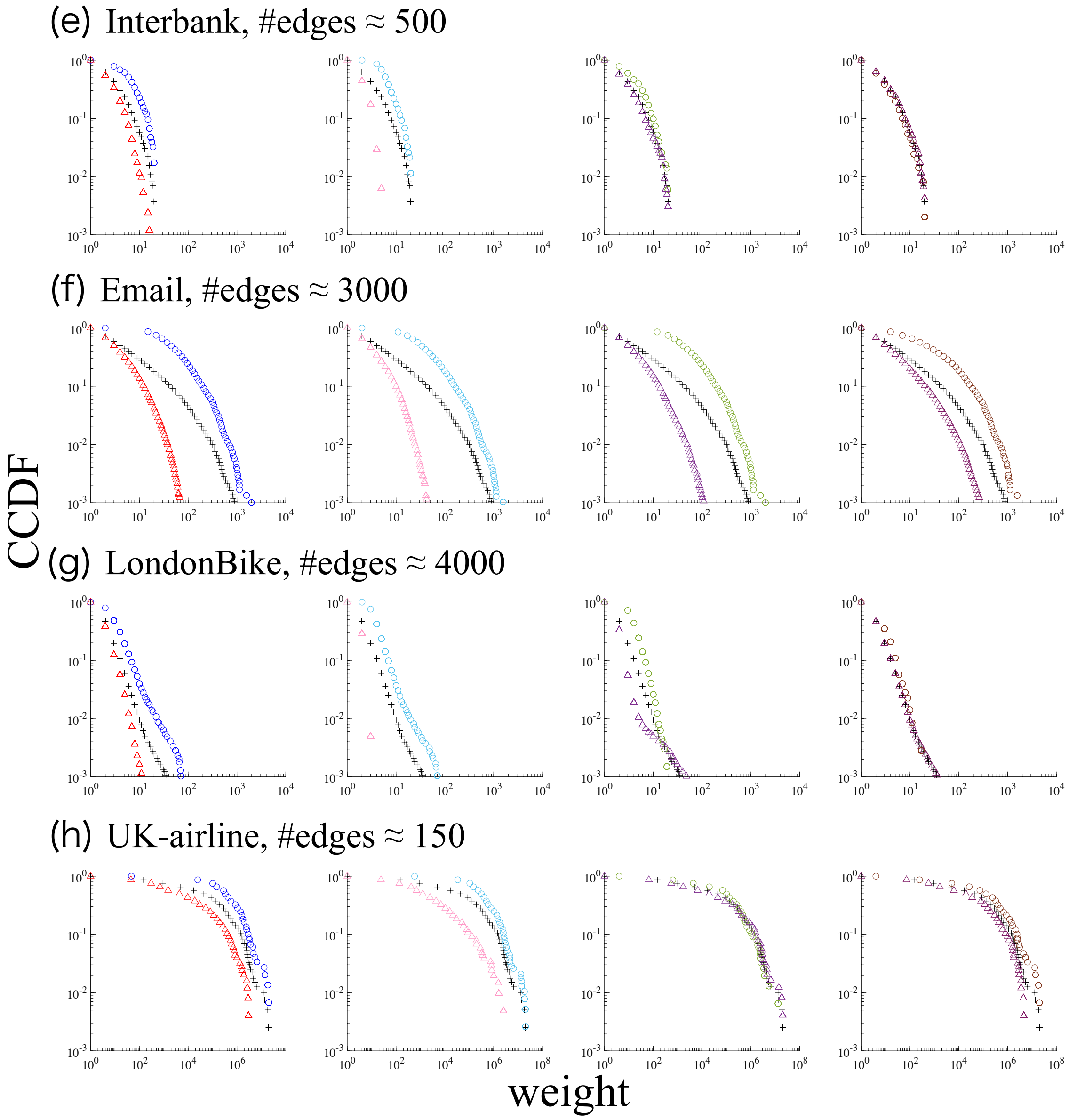
(e) Interbank, #edges ≈ 500
(f) Email, #edges ≈ 3000
(g) LondonBike, #edges ≈ 4000
(h) UK-airline, #edges ≈ 150
CCDF
weight

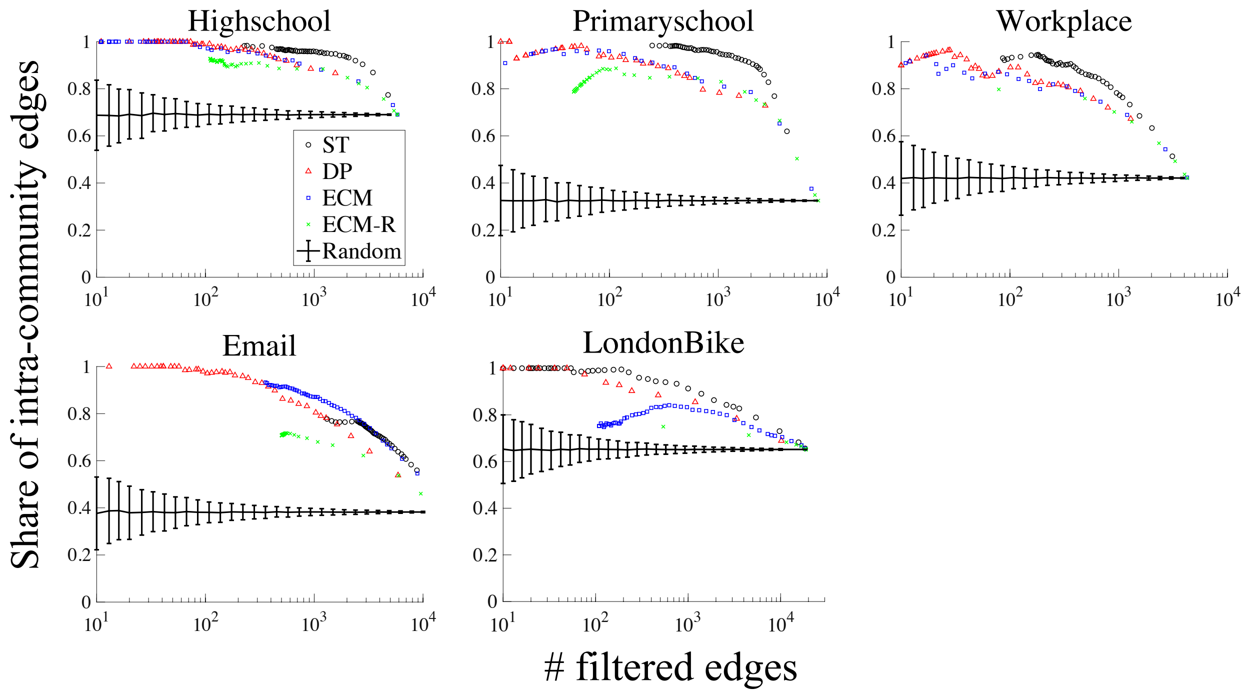

Supplementary Figure 14. Share of intra-community edges among the significant edges, as a function of the number of such edges, for various filters. We consider the networks with $Q > 0.3$. In addition to DP, ECM, ECM-R and ST we show here a random filter selecting edges at random. Error bar denotes the standard deviation calculated over 1,000 runs of random filtering.

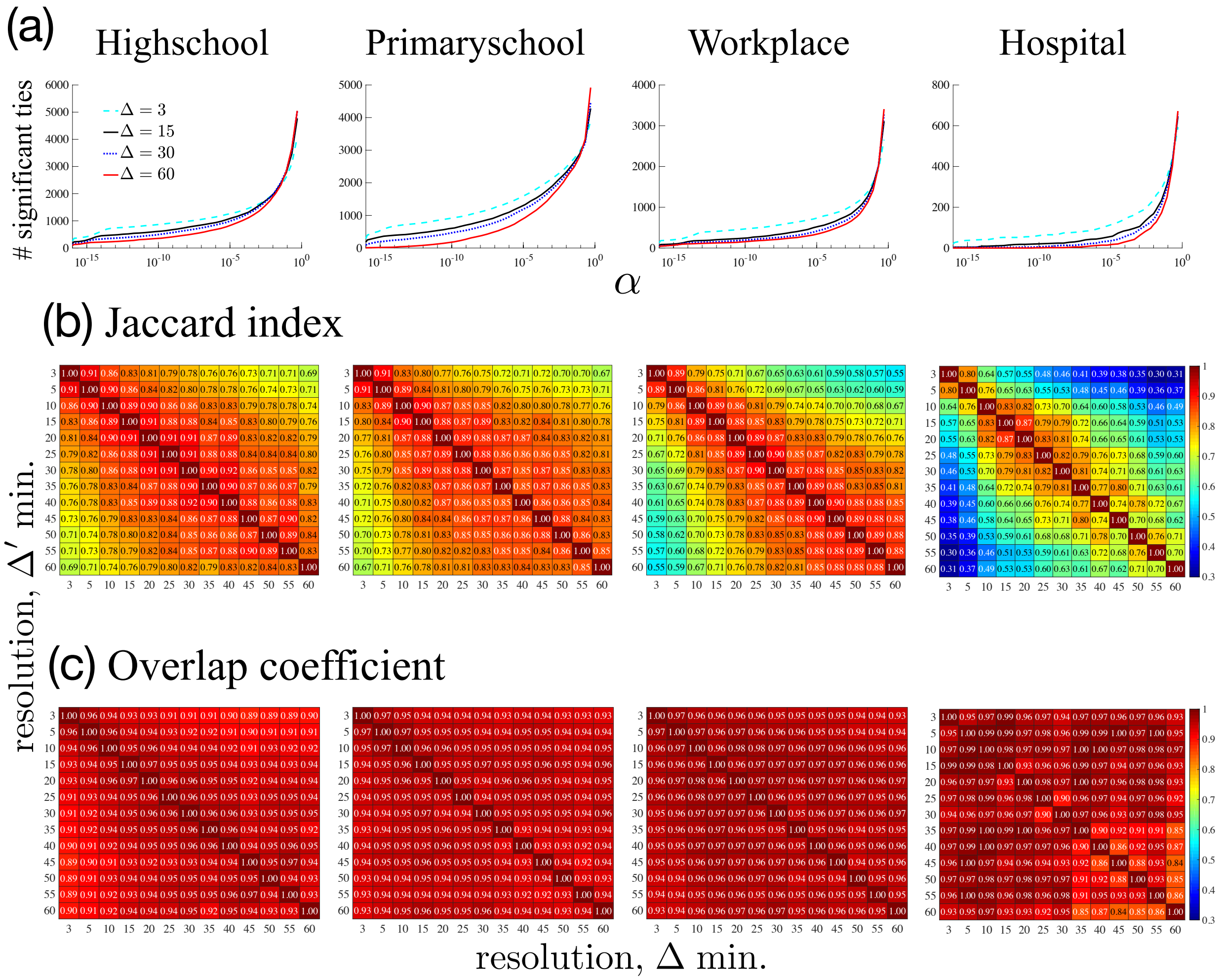

Supplementary Figure 15. Significant ties at different temporal resolutions. (a) Number of significant ties at four different resolutions: $\Delta = \{3, 15, 30, 60\}$. (b) and (c) Heatmap for the overlap of significant ties at different temporal resolutions. In panel (b), we consider the Jaccard index defined by $J(I^{\Delta}_{\mathrm{ST}}, I^{\Delta'}_{\mathrm{ST}}) \equiv |I^{\Delta}_{\mathrm{ST}} \cap I^{\Delta'}_{\mathrm{ST}}| / |I^{\Delta}_{\mathrm{ST}} \cup I^{\Delta'}_{\mathrm{ST}}|$, where I^{Δ}_{ST} denotes the set of significant pairs detected at temporal resolution Δ minutes. In panel (c) we show instead the Overlap coefficient, or Szymkiewicz–Simpson coefficient, defined by $S(I^{\Delta}_{\mathrm{ST}}, I^{\Delta'}_{\mathrm{ST}}) \equiv |I^{\Delta}_{\mathrm{ST}} \cap I^{\Delta'}_{\mathrm{ST}}| / \min(|I^{\Delta}_{\mathrm{ST}}|, |I^{\Delta'}_{\mathrm{ST}}|)$. In (b) and (c), the significance level is set at $\alpha = 10^{-3}$. The difference between the two heatmaps indicates that the main difference stemming from temporal resolutions is just the number of detected pairs; namely, the set of significant ties detected at higher resolutions includes the significant ties detected at lower resolution.

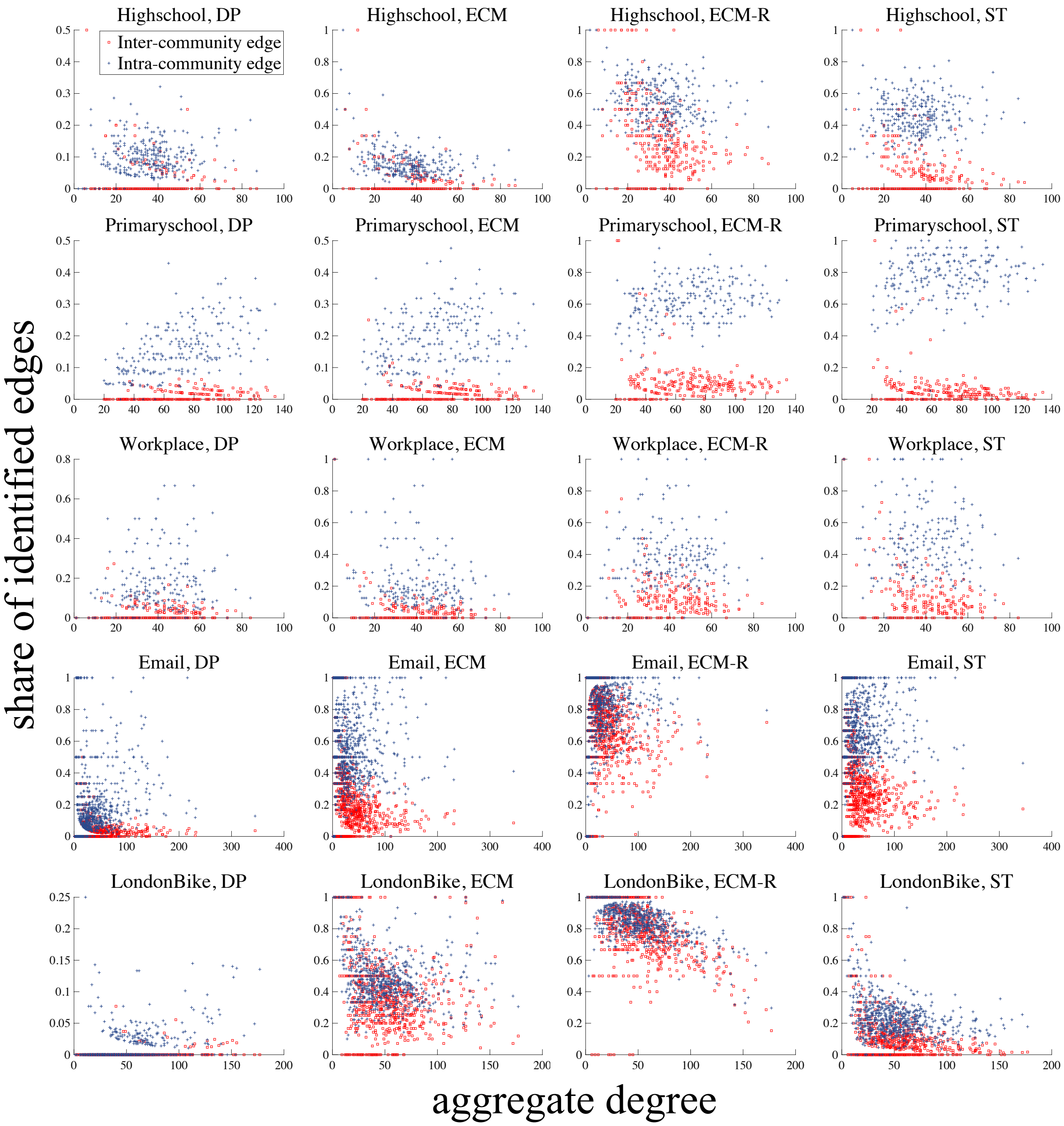

Supplementary Figure 16. Share of filtered edges against aggregate degree in networks with community structure. The red squares (resp. the blue crosses) represent the share of significant inter-community (resp. intra-community) edges among all the inter-community (resp. intra-community) edges emanating from a node. Here $\alpha = 0.01$ The share of significant edges is generally larger among intra-community edges than among inter-community edges.

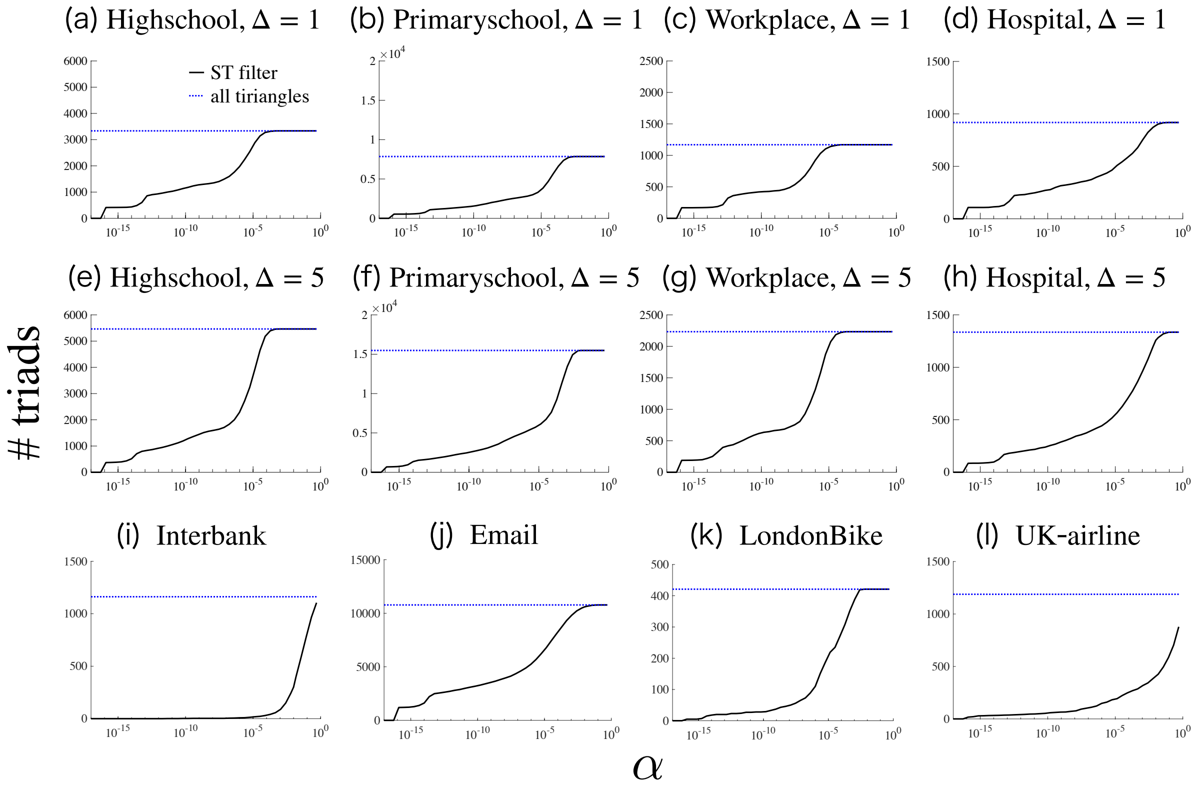

Supplementary Figure 17. Number of significant triads vs. the significance level α, for different data sets and temporal resolutions.

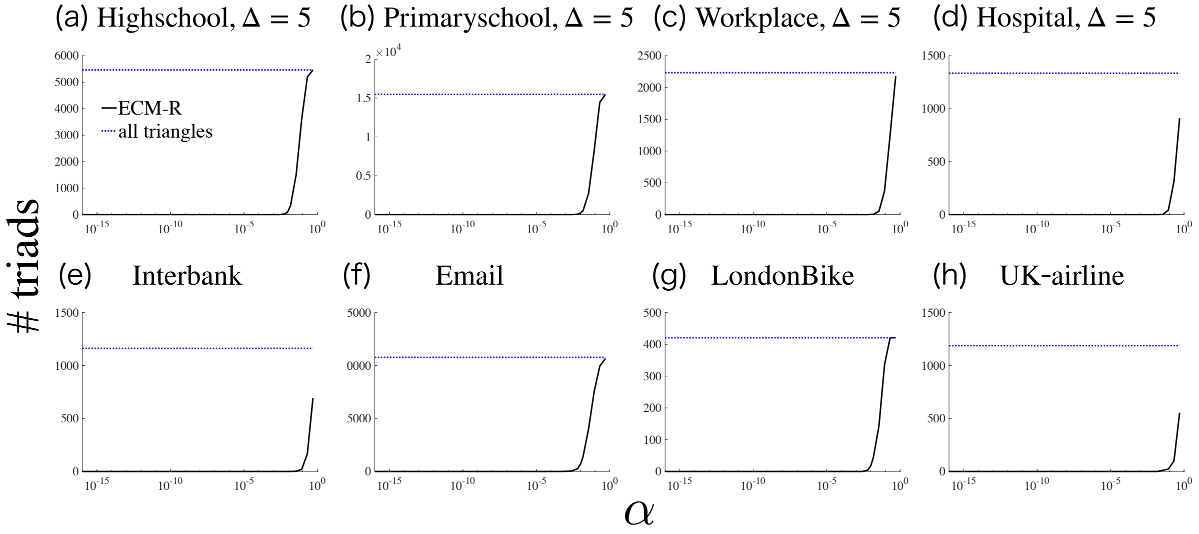

Supplementary Figure 18. Number of triads formed by three ECM-R edges vs. the significance level α. A triad is here regarded as "significant" if the triad is formed by three ECM-R significant edges in at least one snapshot.

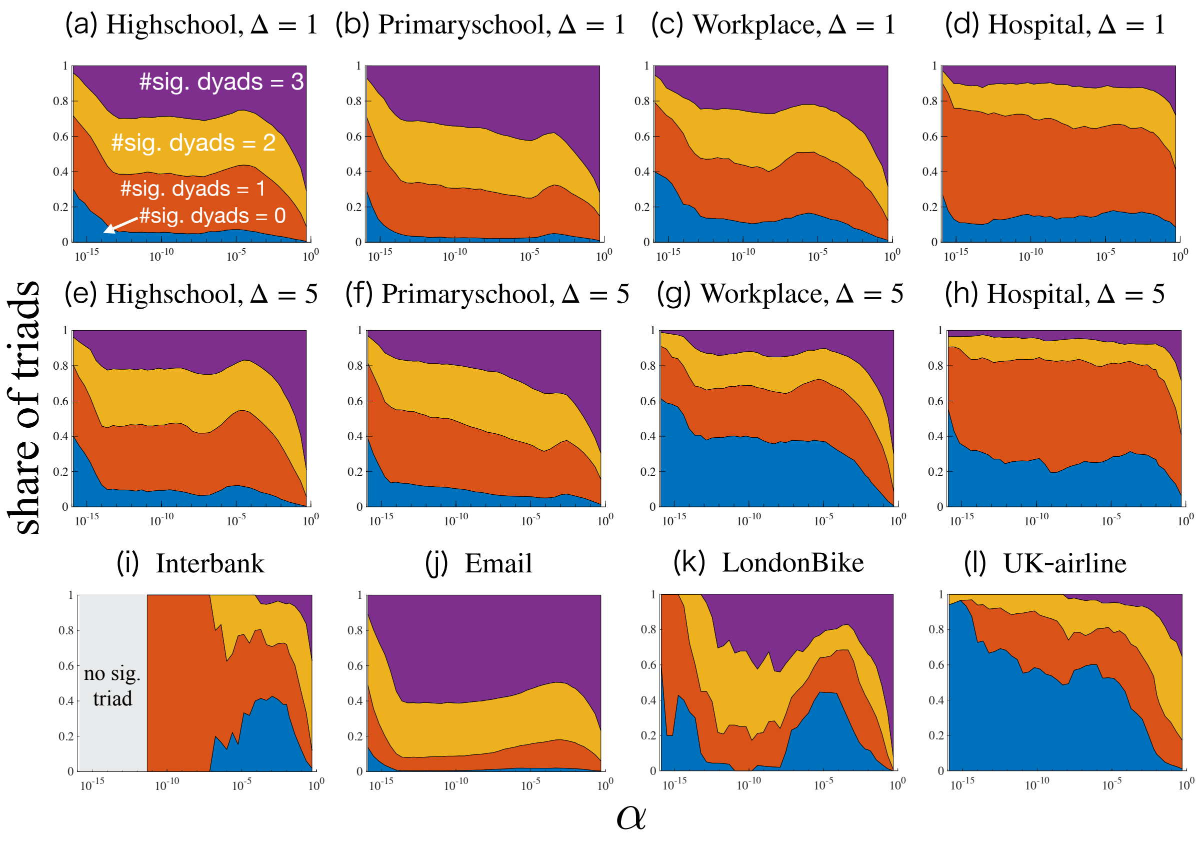

Supplementary Figure 19. Share of triangles with a given number of dyadic significant ties, vs. the filtering level α, for various data sets and temporal resolutions.

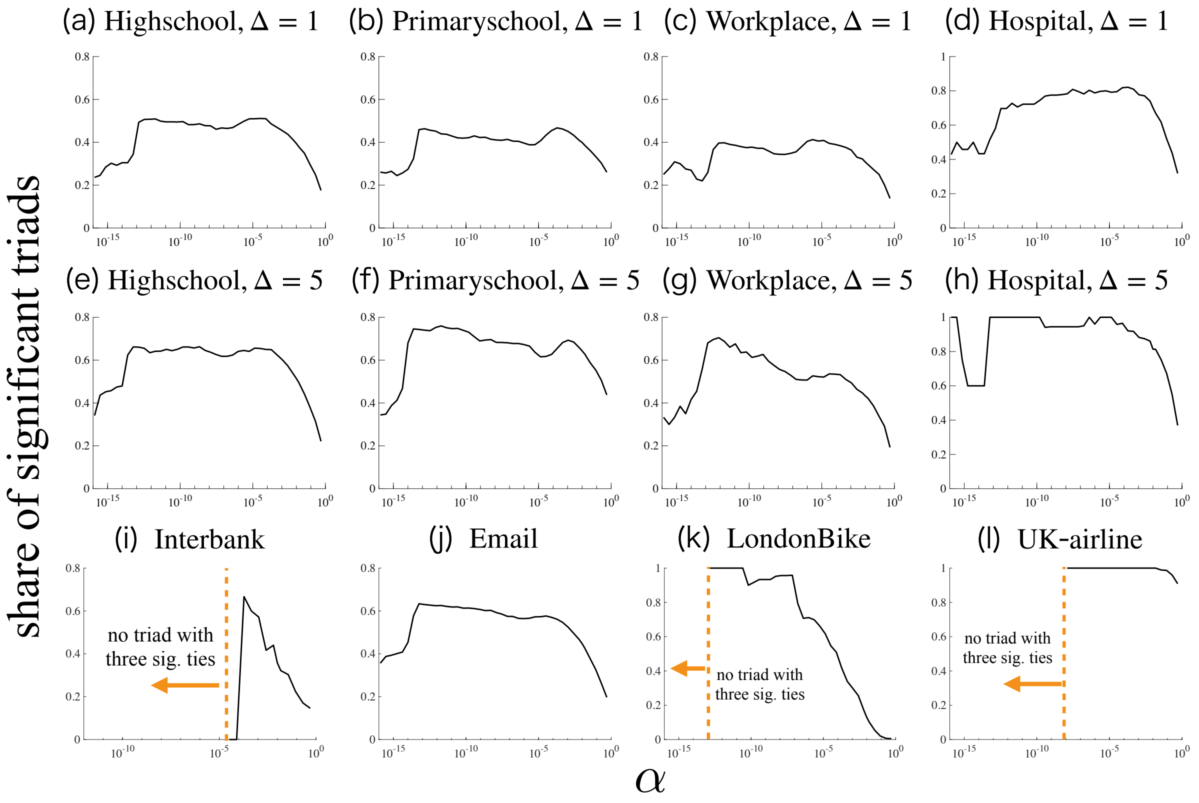

Supplementary Figure 20. Share of significant triads among the triangles composed by three significant ties vs. the filtering level α, for various data sets and temporal resolutions.

Supplementary References

1. Le Cam, L. An approximation theorem for the Poisson binomial distribution. *Pac. J. Math.* **10**, 1181–1197 (1960).
2. Barbour, A. & Eagleson, G. Poisson approximation for some statistics based on exchangeable trials. *Adv. Appl. Probab.* **15**, 585–600 (1983).
3. Steele, J. M. Le cam's inequality and Poisson approximations. *The Am. Math. Mon.* **101**, 48–54 (1994).
4. Serrano, M. Á., Boguná, M. & Vespignani, A. Extracting the multiscale backbone of complex weighted networks. *Proc. Natl. Acad. Sci. USA* **106**, 6483–6488 (2009).
5. Gemmetto, V., Cardillo, A. & Garlaschelli, D. Irreducible network backbones: unbiased graph filtering via maximum entropy. *arXiv:1706.00230* (2017).
6. Squartini, T. & Garlaschelli, D. Analytical maximum-likelihood method to detect patterns in real networks. *New J. Phys.* **13**, 083001 (2011).
7. Mastrandrea, R., Squartini, T., Fagiolo, G. & Garlaschelli, D. Enhanced reconstruction of weighted networks from strengths and degrees. *New J. Phys.* **16**, 043022 (2014).
8. Squartini, T., Mastrandrea, R. & Garlaschelli, D. Unbiased sampling of network ensembles. *New J. Phys.* **17**, 023052 (2015).
9. https://jp.mathworks.com/matlabcentral/fileexchange/46912-max-sam-package-zip.
10. Zhao, K., Stehlé, J., Bianconi, G. & Barrat, A. Social network dynamics of face-to-face interactions. *Phys. Rev. E* **83**, 056109 (2011).
